# Melatonin Improves Laying Performance by Enhancing Intestinal Amino Acids Transport in Hens

**DOI:** 10.3389/fendo.2018.00426

**Published:** 2018-07-30

**Authors:** Lijuan Liu, Siyu Zhang, Jiayang Bao, Xiaowen He, Danni Tong, Cong Chen, Qingxiang Ying, Qing Zhang, Caiqiao Zhang, Jian Li

**Affiliations:** ^1^Department of Veterinary Medicine, College of Animal Sciences, Zhejiang University, Hangzhou, China; ^2^Yanping Bureau of Animal Husbandry, Veterinary & Aquatic Products, Nanping, China; ^3^Nanping Bureau of Agriculture, Nanping, China

**Keywords:** melatonin, laying, intestine, amino acids, transporter, hens

## Abstract

The high concentration of melatonin (MEL) in the intestinal mucosa suggests that it has a special physiological function in intestine. In hens, previous studies have shown that MEL treatment promoted egg-laying performance. Considering the importance of amino acids (AA) for egg formation, we hypothesized that MEL may enhance the intestinal absorption of AA from the feed, thus promoting egg laying performance. In this study, we supplemented the hens with MEL for two consecutive weeks. The results showed that, compared with control group, feeding with 0.625 mg MEL/kg diets gave rise to higher egg laying rate (by 4.3%, *P* = 0.016), increased eggshell thickness (by 16.9%, *P* < 0.01) and albumen height (by 4.5%, *P* = 0.042). Meanwhile, feeding with 0.625 and 2.5 mg MEL/kg diets could significantly increase serum levels of aspartic acid, threonine, serine, glutamic acid, glycine, alanine, isoleucine, leucine, tyrosine, phenylalanine, lysine, histidine, arginine, and proline. Furthermore, a 0.625 mg MEL/kg diets could significantly increase the expression of *PepT1* (by 3949.9%), *B*^0^*AT* (by 6045.9%)*, b*^0, +^*AT* (by 603.5%), and *EAAT3* (by 412.7%) in the jejunum. Additionally, in the cultured intestinal crypt “organoids,” treatment with 0.5 μM MEL could significantly enhance the expression of *PepT1, b*^0, +^*AT* and *EAAT3* mRNAs by 35.4%, 110.0%, and 160.1%, respectively. Detection of MEL concentration in serum and intestinal fluid suggested that lower dosage of MEL feeding was mainly acted on intestine locally, and further increased intestinal antioxidases (*GPx-3, SOD-1* or *PRDX-3*) mRNA expression. Taken together, we demonstrated that MEL feeding in laying hens could locally promote the expression and function of AA transporter in small intestine by up-regulating antioxidases expression, and finally elevate laying performance.

## Introduction

Melatonin (MEL) is mainly produced by the pineal gland. In vertebrates, the MEL level in the intestinal mucosa exceeds its level in the blood plasma by 100 to 400 fold (1, 2). This has led to the notion that there is a distinct physiological function of MEL in the intestines. The highest serum MEL level occurs at midnight and is lowest at midday. However, the secretion of intestinal MEL by the mucosal enteroendocrine cells seems to occur without any evident circadian pattern (3). Interestingly, increased levels of intestinal MEL appear to be linked to food intake rather than photoperiod (4). Moreover, release of intestinal MEL was also related to the periodicity of food intake (5). Importantly, previous studies have demonstrated that MEL could attenuate lipopolysaccharide-induced chicken's small intestine inflammation (6), promote chicken's intestinal mucosal renewal (7), and improve rat's duodenal barrier functions (8). In the aspect of laying performance, MEL treatment could considerably improve egg weight (9), increase the egg-laying rate and reduce the rate of appearance of sharpei eggs in hens (10). Despite these significant findings, the mechanism of MEL's effects on egg production remains unknown.

During the peak laying period, large amount of egg white, and egg yolk formation requires a plentiful nutrition supply. In the small intestine, protein can only be absorbed in the form of amino acids (AA) or, more marginally, di-peptides, tri-peptides, and. Fisher and Johnson (1956) stated that Arg (arginine), His (histidine), Ile (isoleucine), Leu (leucine), Lys (lysine), Met (methionine), Phe (phenylalanine), Thr (threonine), Trp (tryptophan), and Val (valine) are necessary for protein synthesis during egg formation (11). It was noted that Glu (glutamic acid), is particularly essential for maximum egg production (11). Moreover, 0.2% _L_-Thr supplementation was found to markedly increase laying rate and egg weight (12) and dietary Lys and Ile were necessary for maximum egg production (13). Arg-silicate-inositol complex supplementation increased egg production, egg weight, shell weight, and shell thickness during the laying peak period (14). Interestingly, MEL could restore dextran sodium sulfate (DSS)-induced decreasing of Glu and Cys in mice serum (15). However, whether it was AA transported by enterocytes that mediated the MEL effect on egg laying and egg quality remained unknown.

The absorption of AA is dependent on the activities of AA transporters which are located at the enterocytes. APN (Aminopeptidase N), an exopeptidase that cleaves AA from the N-terminus of peptides, occurs at the brush border membrane of the enterocytes (16). PepT1, a peptide transporter, can transport di- and tri-peptides from intestinal lumen into enterocytes (17). B^0^AT (B^0^-type AA transporter), and b^0, +^AT (b^0, +^-type AA transporter) are responsible for up-taking neutral and cationic AA such as Gly, Ser, Thr, Cys, Tyr, Asn, Gln, His, Lys and Arg (18). EAAT3 (excitatory AA transporter) mainly transports aspartate and glutamate (19). y^+^LAT1 (y^+^L AA transporter), is the transporter of Leu, large hydrophobic neutral AA and cationic AA. At the basolateral membrane of the enterocyte, CAT1 (cationic AA transporter 1) and CAT2 can transport cationic AA from the enterocytes into vascular circulation (20). Before hatching, the expression of *APN, PepT1* and *EAAT3* in the chicken intestine is increased from the embryo 15 (E15) stage to that of E21, while CAT1 expression in intestine increases until E17 and then decreases (21). In the chicken, levels of *PepT1* mRNA were higher in the duodenum while *EAAT3, b*^0, +^*AT, rBAT, B*^0^*AT, LAT1, CAT2*, and *APN* mRNA were higher in the ileum, 14 day posthatching (22). The brush border membrane transporters (such as EAAT3 and PepT1) showed increased expression after hatching in chickens (22–24). Interestingly, MEL injection i.p. could promote *B*^0^*AT* and *EAAT3* mRNAs expression in young chicken's small intestine (7). However, which AA transporter plays a critical role during the promotion of MEL on laying property is unclear.

The expression of AA transporters can be influenced by many adverse factors, including those of pathogenic microorganisms and oxidative impairment. In chickens, *Eimeria praecox* challenge can cause the down-regulation of *CAT1, EAAT3, LAT1*, and *PepT1* in the duodenum, down-regulation of *B*^0^*AT* and *CAT1* in the jejunum, and down-regulation of *LAT1* in the ileum (25). Lipid peroxidation can significantly decrease the transportation of AA by enterocyte brush border membrane vesicles (26). Oxidative injury was also noted to decrease PepT1 transport velocity in Caco-2 cells (27). Of note, antioxidant enzyme cascades GPx (glutathione peroxidase) and SOD (superoxide dismutase) act as the first line of protection in counteracting ROS (reactive oxygen species) generation, antioxidant protein PRDX (peroxiredoxin) could protect cells against oxidative damage from ROS. Previously studies showed that MEL can also act to up-regulate the expression of *SOD-1* and the *GPx-4* gene in immature bovine oocytes (28) or enhance *CAT* (catalase), *GPx, SOD* gene expression in the human skin (29). The antioxidant properties of MEL can, for example, restore menadione induced chicken intestinal calcium absorption alterations (30). However, whether intestinal antioxidant defense system can be enhanced during the promotion of MEL on laying property needs to be clarified.

In the present study we elucidate the effect of MEL on hens laying property and egg quality, and screen the key intestinal AA transporter related to laying performance. We also further evaluate the role and possible pathways of MEL act on the expression and function of laying property related intestinal AA transporters.

## Materials and methods

### Animal husbandry and experimental design

A total of 200 HyLine Brown hens at the peak period of laying were randomly divided into four groups (control, 0.625 mg MEL/kg diets, 2.5 mg MEL/kg diets, and 10 mg MEL/kg diets groups, 50 hens/group). These were fed with basal diets (control group) or basal diets containing MEL at the corresponding 0.625, 2.5 and 10 mg/kg diets, respectively, for 2 weeks. The composition and nutrient level of the basal diets is listed in Table 1. The MEL dosages chosen in the present study was based on previously studies (9, 30, 31) and our preliminary experiments. The hens had *ad libitum* access to feed and water, the ambient temperature was maintained at approximately 20–23°C with the relative humidity maintained at about 60%, and the photoperiod was 16 L (light):8 D (dark). During the period of the experiment, the egg laying rate (number of eggs/50 hens) of each treatment group was recorded daily. At the end of the experiment, 30 eggs from each treatment group were randomly collected to assess egg quality parameters. The eggs were weighed and then the eggshell thickness and albumen height were measured with the digital egg tester (DET6000, NABEL Co., Ltd, Kyoto, Japan). For measuring egg protein quality, Haugh units (HU) were calculated by 100 × log ((albumen height) + 7.57–1.7 × (egg weight)∧0.37), which has been accepted for use by the USDA-Agricultural Marketing Service (USDA, 2000).

**Table 1:** Composition and nutrient level of the basal diets.

**Ingredients**	**Content (%)**	**Nutrient**	**Content (per kg diets)**
Corn	61.7	Met	0.11–0.24%
Soybean meal	25.0	Lys	0.22–0.30%
Limestone	8.3	Vitamin mixture	≥58 mg
Additive premix	5	Ca	0.25–1.0%
		P	0.09–0.25%
		Cu	7–21 mg
		Fe	80–650 mg
		Mn,	60–150 mg
		Zn	60–150 mg
		Se	0.1–0.3 mg
		I	0.3–0.9 mg
		NaCl	0.2–0.45%
		Choline chloride	0.28 g

### Sampling

At the end of the experiment (10:00 at the 14th experimental day), five hens per treatment group were randomly chosen for sampling. Blood of these hens were withdrawn by cardiac puncture to obtain serum sample. After that, hens were killed by cervical bleeding post-anesthesia. Intestinal contents were collected from duodenum, spun down at 3000 g for 10 min, and then the supernatant of intestinal fluid were aliquoted and frozen at –20°C until analyzing. Then, the duodenum, jejunum and ileum of each hen was collected and cleaned using PBS (phosphate buffer saline, pH 7.4), then frozen with liquid nitrogen until used for analysis. The blood was placed on 37°C for 2 h then centrifuged at 1000 × g for 10 min to obtain serum, and stored at –70°C until use for analysis. This study was carried out in accordance with the *Guiding Principles for the Care and Use of Laboratory Animals of Zhejiang University*. The experimental protocols were approved by the *Committee on the Ethics of Animal Experiments of Zhejiang University* (No.: ZJU2015-156-12).

### Screening of laying property related intestinal AA transporters

For the identification of which intestinal AA transporter is closely related to laying property, five female Hyline chickens were randomly collected from one of the four stages to analyze mRNA expression of transporters (PepT1, B^0^AT, b^0, +^AT, LAT1, y^+^LAT2, CAT1, and EAAT3) in small intestine by RT-PCR. The four stages including just hatching (1 days old), pre-laying period (about 100 days old), laying peak period (about 300 days old) and post-laying period (about 600 days old). These preliminary screening results were further verified in small intestine of hens of the same age (about 300 days old) but with different laying traits (hens with high laying performance (Hens-H), hens with low laying performance (Hens-L) and broilers) by qRT-PCR.

### RNA extraction, RT-PCR, and quantitative real-time PCR

Trizol reagent (Invitrogen Co., Carlsbad, CA, USA) was used to extract total RNA from small intestine. The cDNA was generated from 2 μg of RNA by using the SuperScript First-Strand Synthesis System (Fermantas, Glen Burnie, MD, USA). PCR amplification was performed on a 20 μl volume containing 2 μl cDNA. For Quantitative Real-time RT-PCR (qRT-PCR), 2 μl cDNA template, 400 nM primers, 0.4 μl ROX reference dye II, and 10 μl SYBR Premix Ex Taq (TaKaRa Bio Inc., Shiga, Japan) were mixed gently and reacted as follows: 95°C for 10 s, 40 cycles of 95°C 5 s followed by 60°C 34 s. All samples were repeated in triplicate, and all experiments were performed twice. All samples were normalized with GAPDH using the comparative cycle threshold method (2^−[Δ]^^[Δ]*Ct*^). The sequences of the primers were listed in Table 2.

**Table 2:** Primers for qRT-PCR analysis.

**Genes**	**Primer sequences (5′-3′)**	**Accession No**.	**Product (bp)**
*PepT1* (SLC15A1)	F: GATCACTGTTGGCATGTTCCT	NM_204365	146
	R: CATTCGCATTGCTATCACCTA		
*B^*O*^AT* (SLC6A19)	F: AATGGGACAACAAGGCTCAG	XM_419056	165
	R: CAAGATGAAGCAGGGGGATA		
*b^*O*, +^AT* (SLC7A9)	F: TATTTCACCGTAATGACTTCAAC	NM_001199133	112
	R: AGGCCACAAAGAGAGGTATTA		
*LAT1* (SLC7A5)	F: TGCCTATGGAGGATGGAAT	NM_001030579.2	150
	R: GTGGACAGGGTTGTGAAGTA		
*y^+^LAT1* (SLC7A7)	F: CCCTGACAGTCTGAGTTTGAT	XM_418326	143
	R: AAAGCCAGTAGTTGAAGCAGT		
*y^+^LAT2* (SLC7A6)	F: ACCCAAAGGAGTTCTCATCT	XM_015292302.1	115
	R: AGTGGTTCCAAGTTCAGCAT		
*CAT1* (SLC7A1)	F: ACCAACAGCCCATTACCCAA	NM_001145490	105
	R: GCCAAGGAGACTCGTAGAAA		
*CAT2* (SLC7A2)	F: TATCCTGACTTCTTTGCCGTATTC	NM_001199102	145
	R: TCACAAAACCAGAAATCATAACGA		
*EAAT3* (SLC1A1)	F: AAAATGGGAGACAAAGGACAA	XM_424930	159
	R: ACGAAAGATTTCCCAGTCCTC		
*GPx-3*	F: AGGAGTACATCCCCTTCCGA	NM_001163232.2	124
	R: TAGGGCCCCAGCTCATTTTG		
*SOD-1*	F: GGCAATGTGACTGCAAAGGG	NM_205064.1	133
	R: CCCCTCTACCCAGGTCATCA		
*PRDX-3*	F: TGGATAAATACCCCGCGCAA	XM_426543.5	126
	R: TCTCAGTGCAATGCCAGGTC		
*GAPDH*	F: GATGGGTGTCAACCATGAGAAA	NM_204305.1	116
	R: CAATGCCAAAGTTGTCATGGA		

### Serum AA analysis

Serum samples were acid hydrolyzed with 1.0 mL 6 mol/L HCl in vacuum-sealed hydrolysis vials at 110°C for 22 h. After centrifugation, the supernatant was then diluted with 0.02 mol/L hydrochloric acid. After filtering through a Millipore membrane (0.22 μm), the content of AA were analyzed using Amino Acid Analyzer (L-8900 Hitachi-hitech, Japan).

### Crypt isolation and culture

Isolation and culture of chicken's intestinal crypts was performed base on the protocol previously published (32). Briefly, clean jejunum of 2-week-old Hyline hens were cut into 2–4 mm^3^ pieces and shaken gently in 2 mM cold EDTA (ethylenediaminetetraacetic acid, pH 7.4). After passing through a 70-μm nylon cell strainer (352360, Corning, NY, USA), the crypts were purifying by centrifugation and re-suspended. Successively, the purified crypts were then mixed with Matrigel (354277, Corning, NY, USA), dropped into 24-well flat-bottom plate, administrated with complete culture medium, and cultured at 38°C in a 5% CO_2_ atmosphere. All these wells were divided into four groups which were added MEL (M5250, Sigma, St. Louis, MO, USA) at different concentrations (0, 0.25, 0.5, and 1 μM) from the beginning to the fifth day of culture. The dosage of MEL treatment was based on our preliminary experiments. After dissolving Matrigel by Cell Recovery Solution (354253, Corning, NY, USA), the crypts “organoids” were collected and spun down. Then, after discarding supernatant, Trizol reagent were used to extract total RNA by protocol above-mentioned.

### Detection of MEL concentration in serum and intestinal fluid

High performance liquid chromatography (HPLC) analysis was performed to detect MEL concentrations in serum and intestinal fluid. Briefly, 200 μl of serum or intestinal fluid was mixed with 1 ml methanol, shaken for 20 min and centrifuged at 12000 × g for 10 min. The supernatants were then evaporated until dry and dissolved in 1 ml ethyl acetate, shaken for 20 min and centrifuged at 12000 × g for 10 min. The supernatants were re-dissolved in 200 μl HPLC mobile phase (acetonitrile: water = 25:75, v/v, pH 5.1). After filtering through a Millipore membrane (0.22 μm), the samples (20 μl) were injected into a Ultimate AQ-C_18_ column (5 μm, 4.6 × 250 mm), and then separated by chromatographic system (LC-20, Shimadzu Corp., Kyoto, Japan) at a flow rate of 1 ml/min. The RF-20A fluorescence detector (Shimadzu Corp., Kyoto, Japan) was set to excitation/emission wavelength of 285/345 nm.

### Data analysis

The data were expressed as the means ± SEM and analyzed by One-Way ANOVA using SPSS 16.0 (SPSS Inc., Chicago, IL, USA). Statistical differences among different groups were evaluated by LSD (Least Significant Difference) Post Hoc Multiple Comparisons test. The significance level was set at *P* < 0.05.

## Results

### Effect of MEL on egg laying rate and egg quality

**Figure 1:**
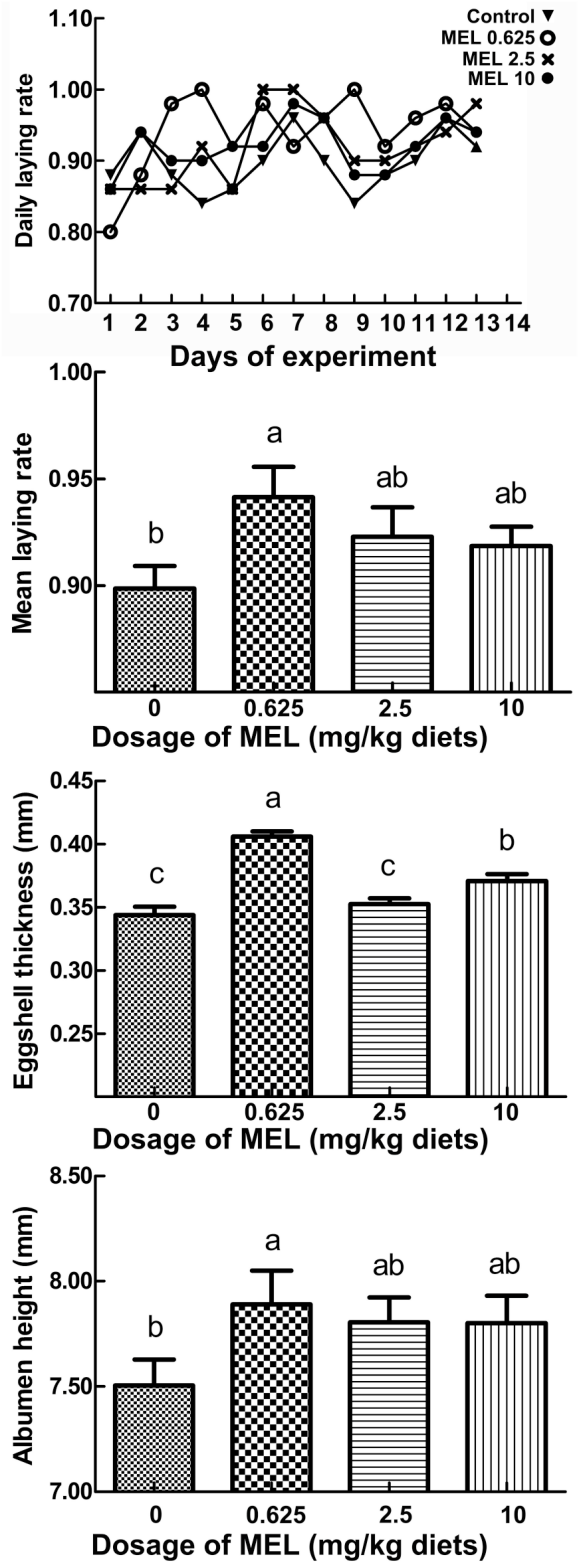
The effect of MEL feeding on laying performance and egg quality. Laying hens were fed daily with diets containing 0, 0.625, 2.5, and 10 mg MEL/kg, respectively, for 2 weeks. Thereafter, laying performance and egg quality were analyzed. The daily laying rate of each group was calculated as “number of eggs/50 hens.” The mean laying rate of each group was the average of the daily laying rate during the experimental period. Values with no common letters are significantly different (*P* < 0.05).

The daily laying rate of 0.625 mg MEL/kg diets groups maintains at a higher level throughout the experiment period (Figure 1). Compared with control group, the mean egg laying rate in the 0.625, 2.5, and 10 mg MEL/kg diets groups was increased by 4.3% (*P* = 0.016), 2.4% (*P* = 0.163), and 2.0% (*P* = 0.250), respectively during the MEL feeding period (Figure 1). For analysis of the effect of MEL on egg quality, the eggshell thickness, albumen height, and HU of the eggs were assayed. The results (Figure 1) showed that eggshell thicknesses of the 0.625, 2.5, and 10 mg MEL/kg diets groups were higher than those of the control group by 16.9% (*P* < 0.01), 1.5% (*P* = 0.465), and 6.8% (*P* < 0.01), respectively. Simultaneously, albumen heights for the 0.625, 2.5, and 10 mg MEL/kg diets groups were higher at the ratios of 4.5% (*P* = 0.042), 3.4% (*P* = 0.110), and 3.3% (*P* = 0.111) of those of control group, respectively. However, the HU of eggs of the 0.625, 2.5, and 10 mg MEL/kg diets groups were higher only by 0.1–1.8% and without statistical difference.

### Effect of MEL on serum AA levels

After feeding MEL to laying hens for 2 weeks, the hens serums AA were measured. As shown in Table 3, compared with control group, the 0.625 and 2.5 mg MEL/kg diets groups presented statistically significant higher Asp (by 220.4–233.3%, *P* < 0.01), Thr (by 80.0–118.3%, *P* < 0.01), Ser (by 90.1–111.3%, *P* < 0.01), Glu (242.8–274.2%, *P* < 0.01), Gly (by 363.2–368.4%, *P* < 0.01), Ala (by 292.9–308.9%, *P* < 0.01), Ile (116.6–116.7, *P* < 0.01), Leu (by 198.9–225.5%, *P* < 0.01), Tyr (by 98.2–105.4%, *P* < 0.01), Phe (by 209.9–215.5%, *P* < 0.01), Lys (by 217.8–242.5%, *P* < 0.01), His (by 373.1–380.7%, *P* < 0.01), Arg (by 171.6–182.1%, *P* < 0.01), and Pro (by 313.6–327.3%, *P* < 0.01). In addition, the 10 mg MEL/kg diets group presented higher Ser (by 88.7%, *P* < 0.01), Glu (190.3%, *P* < 0.01), Cys (by 125.0%, *P* < 0.01), Leu (116.0%, *P* < 0.01), Tyr (by 89.3%, *P* < 0.01), Phe (by 46.5%, *P* < 0.01), and His (188.5%, *P* < 0.01) than those of the control group.

**Table 3:** Effect of MEL feeding on serum AA levels in laying hens (*n* = 5).

**AA (g/100g protein)**	**Control**		**Melatonin feeding (mg/kg diets)**					
			**0.625**		**2.5**		**10**	
	**Mean**	**SEM**	**Mean**	**SEM**	**Mean**	**SEM**	**Mean**	**SEM**
Asp	0.93	0.04^b^	3.10	0.18^a^	2.98	0.18^a^	1.47	0.13^b^
Thr	0.60	0.02^b^	1.31	0.18^a^	1.08	0.09^a^	1.03	0.05^ab^
Ser	0.71	0.04^b^	1.50	0.11^a^	1.35	0.06^a^	1.34	0.09^a^
Glu	1.24	0.04^b^	4.64	0.26^a^	4.25	0.41^a^	3.60	0.07^a^
Gly	0.38	0.02^b^	1.76	0.12^a^	1.78	0.11^a^	0.68	0.19^b^
Ala	0.56	0.04^b^	2.20	0.56^a^	2.29	0.42^a^	1.27	0.05^ab^
Cys	0.16	0.01^b^	0.14	0.01^b^	0.17	0.06^b^	0.36	0.03^a^
Val	0.82	0.05^b^	1.40	0.22^ab^	1.48	0.14^a^	1.14	0.10^ab^
Met	0.37	0.07	0.46	0.06	0.71	0.11	0.67	0.11
Ile	0.72	0.17^b^	1.56	0.10^a^	1.56	0.17^a^	1.00	0.07^ab^
Leu	0.94	0.10^c^	3.06	0.13^a^	2.81	0.32^ab^	2.03	0.04^b^
Tyr	0.56	0.01^b^	1.11	0.06^a^	1.15	0.05^a^	1.06	0.10^a^
Phe	0.71	0.01^c^	2.24	0.07^a^	2.20	0.06^a^	1.04	0.05^b^
Lys	0.73	0.07^b^	2.50	0.09^a^	2.32	0.27^a^	1.24	0.16^b^
His	0.26	0.01^c^	1.23	0.03^a^	1.25	0.06^a^	0.75	0.06^b^
Arg	0.67	0.06^b^	1.82	0.14^a^	1.89	0.14^a^	0.90	0.09^b^
Pro	0.44	0.03^b^	1.82	0.10^a^	1.88	0.16^a^	0.53	0.03^b^

a-d *Values with no common letters are significantly different (P < 0.05) within one AA*.

### Screening of intestinal AA transporters which is related to laying property

**Figure 2:**
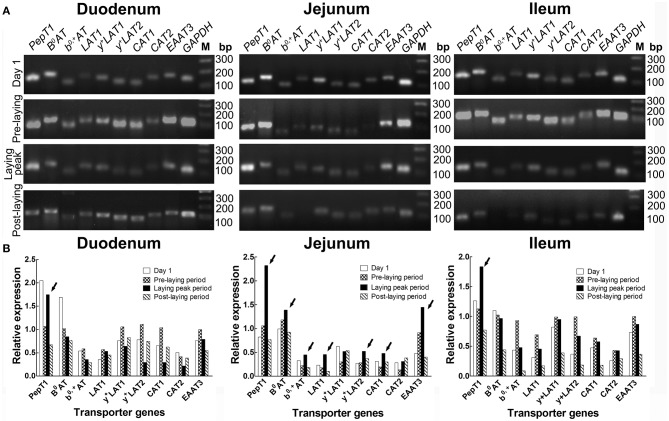
Screening by RT-PCR of intestinal AA transporters, which are closely related to laying performance, at different ages. **(A)** RT-PCR results of various AA transporters in duodenum, jejunum and ileum at the stage of 1 days old, pre-laying period (about 100 days old), laying peak period (about 300 days old) and post-laying period (about 600 days old), respectively. **(B)** Histograms representing relative expression of RT-PCR result. The data is expressed as the integral optical density (IOD) of the bands, normalized to the IOD of the corresponding *GAPDH* bands. Arrows indicated AA transporters as highly expressed in the laying peak period.

The RT-PCR screening results (Figure 2) showed that, in the duodenum, *PepT1, B*^0^*AT, b*^0, +^*AT, LAT1*, and *EAAT3* were more highly expressed in laying peak period than in the post-laying period, especially for *PepT1* which was at an ever higher level than that of the pre-laying period (arrows, Figure 2B). In the jejunum, *PepT1, B*^0^*AT, b*^0, +^*AT, LAT1, y*^+^*LAT2, CAT1*, and *EAAT3* levels were higher in laying peak period than in the post-laying period. Among them, *PepT1, B*^0^*AT, b*^0, +^*AT, LAT1, y*^+^*LAT2, CAT1*, and *EAAT3* were even higher than that in the pre-laying period (arrows, Figure 2B). In the ileum, all the transporters detected were higher in the laying peak period than in the post-laying period, moreover, *PepT1* levels were higher than in the pre-laying period (arrows, Figure 2B).

**Figure 3:**
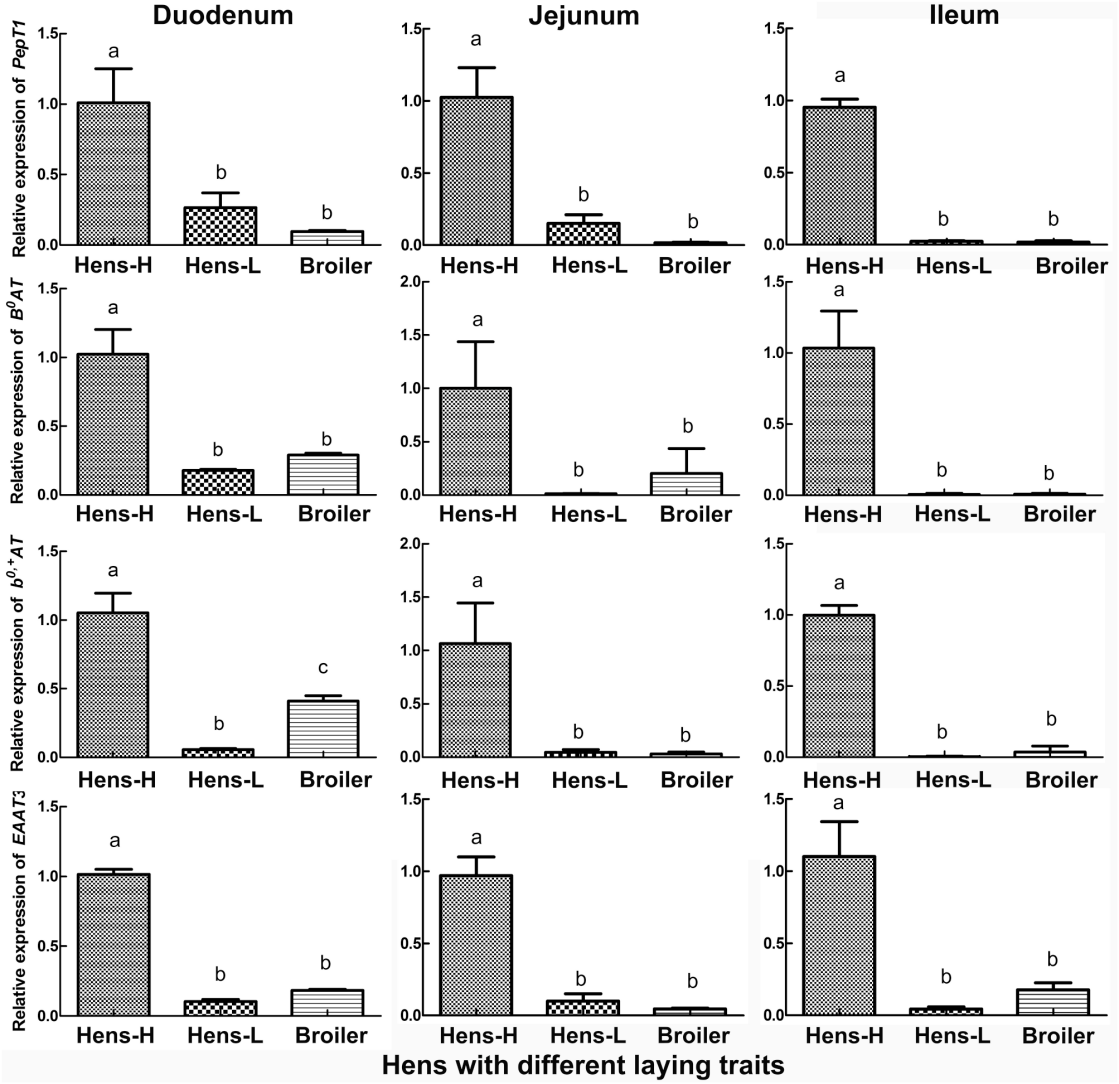
Screening by qRT-PCR of intestinal AA transporters, which are closely related to laying performance, among hens with different productive traits. Hens-H, hens with high laying performance. Hens-L, hens with low laying performance. Values with no common letters are significantly different (*P* < 0.05).

The qRT-PCR verification (Figure 3) in the small intestine of hens with different productive traits (hens-H, hens-L and broilers) showed that throughout the small intestine (duodenum, jejunum and ileum), hens-H presented dramatically higher *PepT1* (281.6–3924.0% higher than hens-L and 961.7–6312.6% higher than broilers), higher *B*^0^*AT* (479.3–1597.9% higher than hens-L and 253.7–1221.6% higher than broilers), higher *b*^0, +^*AT* (1801.6–2097.1% higher than hens-L and 156.9–3363.9% higher than broilers), and higher *EAAT3* (861.2–2414.3% higher than hens-L and 452.2–1977.1% higher than broilers).

### Effect of MEL on transporter mRNA expression in small intestine of laying hens

**Figure 4:**
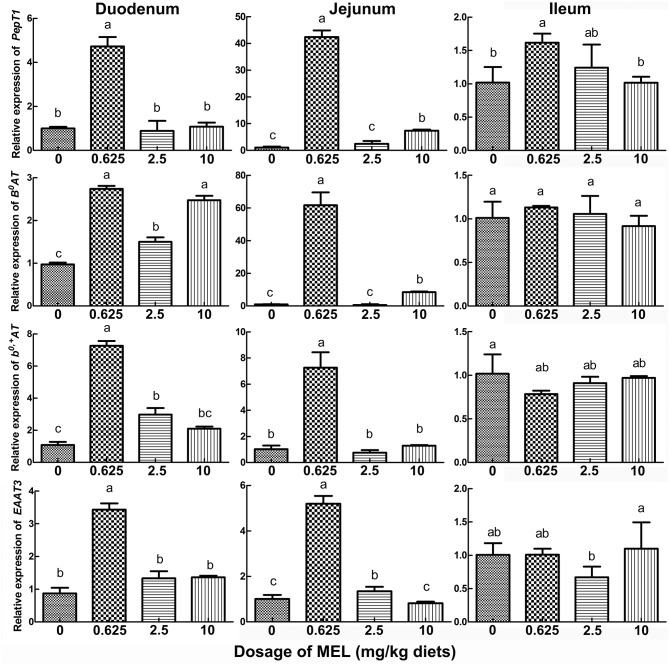
Histograms representing the effect of MEL feeding on the expression of key intestinal AA transporters *in vivo*. Laying hens were daily fed diets containing 0, 0.625, 2.5, and 10 mg MEL/kg, respectively, for 2 weeks. Thereafter, key intestinal AA transporters were analyzed by qRT-PCR. Values with no common letters are significantly different (*P* < 0.05).

In the peak laying period and after MEL (0.625, 2.5, and 10 mg/kg diets) feeding for 2 weeks, *PepT1, B*^0^*AT, b*^0, +^*AT*, and *EAAT3* expressions in small intestine were analyzed. The results (Figure 4) showed that, compared with control group, 0.625 mg MEL/kg diets promoted the expression of *PepT1* (by 374.3%, *P* < 0.01), *B*^0^*AT* (by 182.5%, *P* < 0.01), *b*^0, +^*AT* (by 569.7%, *P* < 0.01), and *EAAT3* (by 293.9%, *P* < 0.01) in the duodenum, promoted the expression of *PepT1* (by 3949.9%, *P* < 0.01), *B*^0^*AT* (by 6045.9%, *P* < 0.01)*, b*^0, +^*AT* (by 603.5%, *P* < 0.01), and *EAAT3* (by 412.7%, *P* < 0.01) in the jejunum, and promoted the expression of *PepT1* (by 58.7%, *P* = 0.018) in the ileum. Moreover, 2.5 mg MEL/kg diets increased the expression of *b*^0, +^*AT* (by 174.5%, *P* < 0.01) in the duodenum and *EAAT3* (by 33.1%, *P* < 0.01) in the jejunum. Furthermore, 10 mg MEL/kg diets treatment elevated the expression of *B*^0^*AT* (by 751.9%, *P* < 0.01) and *PepT1* (by 601.7%, *P* < 0.01) in the jejunum.

### Effect of MEL on transporter transcription in cultured crypt “organoids”

**Figure 5:**
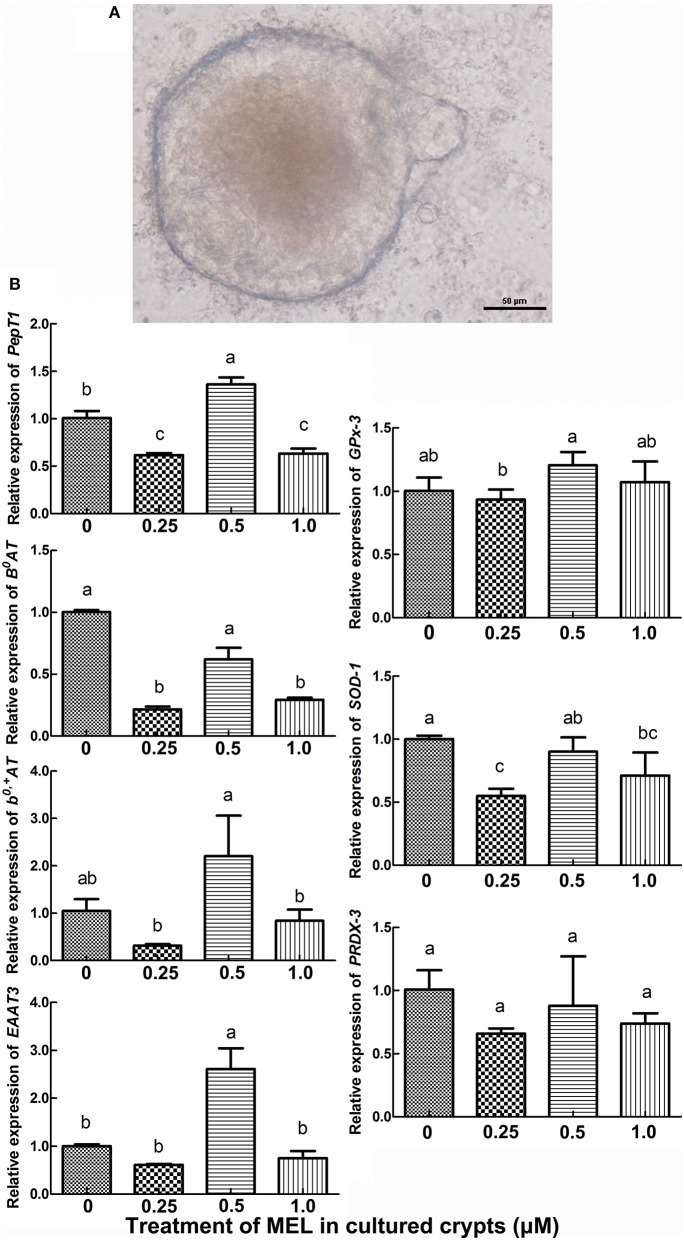
Histograms representing effect of MEL treatment on key intestinal AA transporters and antioxidase mRNA expression *in vitro*. **(A)** *in vitro* cultured chicken crypt “organoids” which were treated with MEL at the concentrations of 0, 0.25, 0.5, and 1 μM for 5 days. Scale bar: 50 μm. **(B)** qRT-PCR result of various AA transporters and antioxidase's mRNA expression in crypt “organoids” after MEL treatment. Values with no common letters are significantly different (*P* < 0.05).

Intestinal stem cells in the crypt can differentiate into various types of epithelial cells, including enterocytes. Hence, *in vitro* cultured crypt “organoids” can be used for the analysis of the effect of MEL on the transcription of intestinal AA transporters. In the present study, we added MEL (0.25, 0.5, and 1 μM) to the chicken's intestinal crypt “organoids” for 5 days (Figure 5A) where qRT-PCR were then used to detect the expression of *PepT1, B*^0^*AT, b*^0, +^*AT*, and *EAAT3*. The results (Figure 5B) showed that, compared with 0 μM MEL group, 0.5 μM MEL treatment enhanced the expression of *PepT1* and *EAAT3* by 35.4% (*P* = 0.028) and 160.1% (*P* = 0.021) respectively. However, *b*^0, +^*AT* at 0.5 μM MEL treatment group was higher than control group by 110.0%, but without statistical significance (*P* = 0.203).

### MEL concentrations in serum and intestinal fluid

**Figure 6:**
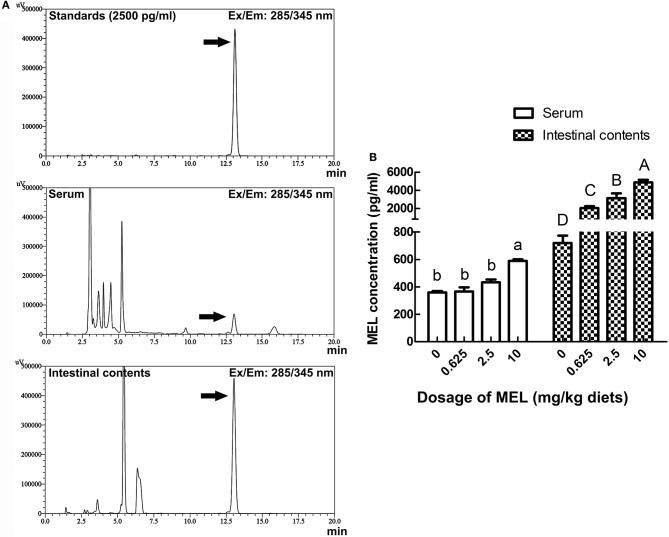
Effects of MEL feeding on MEL concentrations in serum and intestinal fluid by HPLC. **(A)** HPLC result of 2500 pg/ml MEL standards, serum, and intestinal contents. **(B)** Statistical result of MEL concentrations in serum and intestinal fluid of laying hens after MEL feeding. Values with no common letters are significantly different (*P* < 0.05).

For analyzing the acting pathway of MEL, 1 h after MEL feeding at the 14th day of experiment period, concentrations of MEL in serum and intestinal fluid were determined by HPLC. As shown in Figure 6, in serum, MEL concentration in 10 mg/kg diets group (588.5 pg/ml) was significantly higher than the control group (360.1 pg/ml), 0.625 mg/kg diets group (365.5 pg/ml) and 2.5 mg/kg diets group (433.3 pg/ml). However, there has no significant difference amount other groups. Meanwhile, intestinal fluid showed an increase in MEL concentration with increasing MEL dosage in the diets. After MEL feeding, much higher MEL concentrations were observed in the intestinal fluid than that of the control group.

### Effect of MEL on antioxidase gene transcription in crypt “organoids” and the small intestine

**Figure 7:**
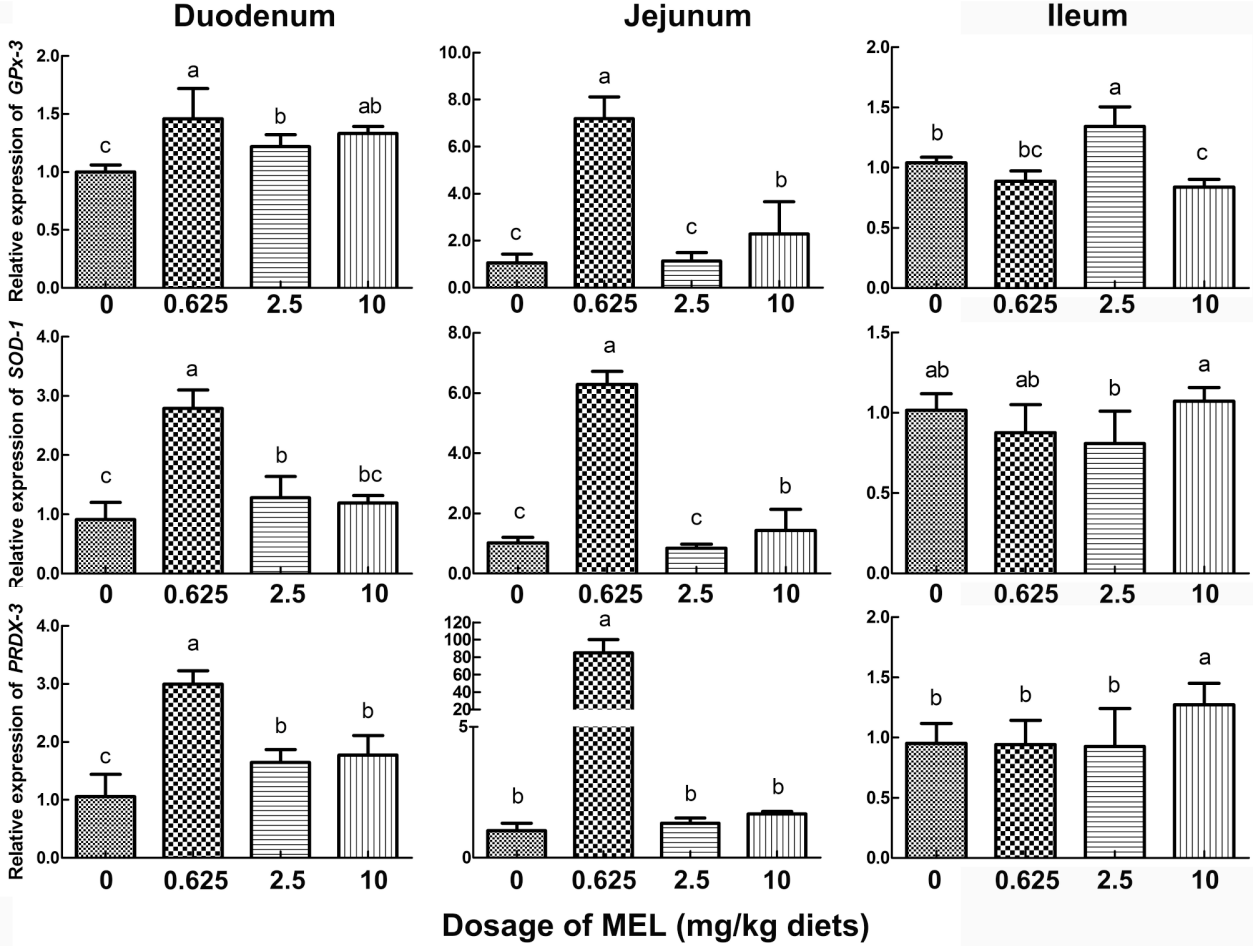
Histograms representing effect of MEL treatment on intestinal antioxidase mRNA expression *in vivo*. Laying hens were fed daily with diets containing 0, 0.625, 2.5, and 10 mg MEL/kg, respectively, for 2 weeks. Thereafter, key antioxidases in small intestine were analyzed by qRT-PCR. Values with no common letters are significantly different (*P* < 0.05).

To further validate the possible pathway of MEL promotion on laying properties, the expression of the antioxidase genes *GPx-3, SOD-1*, and *PRDX-3* were analyzed. In the crypt “organoids” (Figure 5B), *GPx-3* was higher in 0.5 μM MEL treatment group than control group by 20.2% (*P* = 0.076). In the aspect of *in vivo* study, compared with control group, nearly all the MEL dosages could enhance *GPx-3, SOD-1*, and *PRDX-3* expression in the duodenum (Figure 7), especially for 0.625 mg MEL/kg diets group which presented an even higher *GPx-3* (by 45.9%, *P* < 0.01), *SOD-1* (by 205.4%, *P* < 0.01), and *PRDX-3* (by 184.3%, *P* < 0.01). In the jejunum, the 0.625 mg MEL/kg diets remarkably promoted the expression of *GPx-3, SOD-1*, and *PRDX-3* by 579.2% (*P* < 0.01), 517.9% (*P* < 0.01) and 8130.0% (*P* < 0.01) respectively, when compared with control group. However, the antioxidant function of MEL in the ileum remained mild where *GPx-3* had only showed an increased in the 2.5 mg MEL/kg diets group (by 29.0%, *P* < 0.01), and only *PRDX-3* showed an increase in the 10 mg MEL/kg diets group (by 33.8%, *P* < 0.01), when compared with control group.

## Discussion

In this study, feeding the 0.625 mg MEL/kg diets could significantly increase egg laying rate. Similarly, feeding of 0.03–3 mg/kg MEL diets could considerably improve egg weight in laying hens (9). MEL implanted into 470 days old hens could increase the egg-laying rate and reduce the frequency of the occurrence of sharpei eggs (10). Eggshell and albumen quality are important fundamental economic indexes for the egg industry. Here, eggshell thickness and albumen height could be increased by feeding the 0.625 mg MEL/kg diets. In addition, eggshell thickness could also be increased by dietary xylanase (33). Numerous studies have shown that egg albumen height could be increased by dietary supplements such as xylanase (33), vitamins C (34) or tea polyphenols (35). Conversely, increased plasma MEL can elevate hypothalamic gonadotropin-inhibitory hormone (GnIH) mRNA expression in chicken (36) and quail (37). Increased GnIH release acts to inhibit plasma luteinizing hormone (LH) concentrations, which will then repress ovarian function. In the present study, 1 h after MEL feeding, serum MEL level of lower dosage feeding group (0.625 and 2.5 mg/kg diets) were comparable to that of control group and significantly lower than higher dosage feeding group (10 mg/kg diets). These results align well with our finding that a lower dosage of MEL supplement could improve laying performance and egg quality, but that higher dosage of MEL supplementation may suppress the physiological function of the ovary, and subsequently compromise the beneficial effect of MEL on laying property.

For demonstrating whether the improvement of MEL on laying property and egg quality was mediated by intestinal AA transport, we screened the AA transporters which are closely related to laying performance. Compared with pre-laying or post-laying period, *PepT1, B*^0^*AT, b*^0, +^*AT, LAT1, y*^+^*LAT2, CAT1*, and *EAAT3* were highly expressed in the jejunum of hens in laying peak period. Moreover, *PepT1, B*^0^*AT, b*^0, +^*AT*, and *EAAT3* in small intestine of hens-H were notably higher than hens-L and broilers. In as much as most AA absorption takes place in the jejunum (38), our result indicated that PepT1, B^0^AT, b^0, +^AT, and EAAT3 in the jejunum was closely related to laying performance. Interestingly, after MEL treatment, *PepT1, B*^0^*AT, b*^0, +^*AT*, and *EAAT3* mRNA expression in the duodenum and jejunum of laying hens were also substantially elevated, and *PepT1* and *EAAT3* levels in the intestinal crypt “organoids” were also considerably increased. Consistently, MEL i.p. provision could promote intestinal *B*^0^*AT* and *EAAT3* mRNAs expression in young chickens (7), where the transcription of the *B*^0^*AT* gene in the enterocytes is regulated at three different levels involving promoter methylation, histone modification, and opposing transcription factors (39). Similarly, *PepT1* expression in the rat duodenum could be enhanced by zinc glycine chelate supplementation (40). These results indicated that *PepT1, B*^0^*AT, b*^0, +^*AT*, and *EAAT3* expression in the small intestine of laying hens could be up-regulated by MEL.

Egg laying performance can be influenced by AA absorption. Azzam et al. (2011) demonstrated that 0.2% L-Thr supplementation resulted in optimal egg production (12). Adequate dietary Lys and Ile should be provided for maximum hatching egg production (13). The Arg-silicate-inositol complex supplementation in laying hens during the peak laying period improved eggshell quality through improving calcium utilization (14). In addition to Thr, Lys and Arg which have shown above, a lower dosage of MEL (0.625 or 2.5 mg/kg diets) in the present study could dramatically elevate serum Asp, Thr, Ser, Glu, Gly, Ala, Ile, Leu, Tyr, Phe, Lys, His, Arg, and Pro levels in laying hens. These results are almost completely in accordance with the function of MEL-enhanced intestinal *PepT1, B*^0^*AT, b*^0, +^*AT*, and *EAAT3*. Similarly, intestinal *B*^0^*AT* and *EAAT3* mRNAs expression in young chicken could be elevated by MEL (1–10 μg/d) injection (7). MEL (0.2 mg/mL) could also restore DSS-induced decrease of Glu and Cys in mice serum (15). These results indicate that lower dosage of MEL feeding in laying hens could increase the AA levels in serum by enhancing transcription of *PepT1, B*^0^*AT, b*^0, +^*AT*, and *EAAT3* mRNA in the small intestine.

A previous study demonstrated that the physiological dose of MEL in the chicken was between 100 pg/ml and 10 ng/ml (41). In this study, serum MEL concentration in laying hens was about 300–400 pg/ml at midday, and feeding lower MEL dosage (0.625 and 2.5 mg /kg diets) did not increase serum MEL level significantly. However, MEL concentration in the intestinal fluid increased with increasing MEL dosage in diets. These results suggested that the effects of MEL feeding were mainly exerted by acting locally on intestine instead of increasing circulating MEL levels. In the rat, exogenous MEL treatment (1–1000 mg/kg, i.p.) may act at MEL receptors at lower doses (1 or 10 mg/kg) to increase intestinal motor activity (42). Under the treatment of MEL (10^−3^-10^−9^ M) *in vitro*, the ewes' embryos developed well under the lower MEL concentration (10^−9^ M), while the highest MEL concentration decreased their viability by reducing ATP concentration (43). These results suggested that lower dosage of MEL feeding in our study was much advantageous to intestinal physiological function. Many studies have shown that lipid peroxidation (26) or oxidative injury (27) can decrease AA transportation in the enterocytes. We demonstrated that MEL feeding could significantly promote *GPx-3, SOD-1*, and *PRDX-3* expression in the duodenum and jejunum of laying hens. *In vitro*, 0.5 μM MEL could also considerably enhance *GPx-3* expression in crypt “organoids.” These indicated that the promotion of *PepT1, B*^0^*AT, b*^0, +^*AT*, and *EAAT3* expression and function by MEL may be mediated by this improved antioxidant status.

In conclusion, this study revealed that lower dosage (0.625 mg /kg diets) of MEL feeding in laying hens could act locally on intestine, enhance the intestinal antioxidase gene expression, which further promotes the expression of intestinal *PepT1, B*^0^*AT, b*^0, +^*AT*, and *EAAT3* and corresponding AA transportation, and result in improved egg laying performance.

## Author contributions

LL, SZ, CC, QY, QZ, CZ, and JL conceived the experiment(s). LL, SZ, JB, XH, DT, CC, QZ, and JL conducted the experiments; all authors joined the analysis and interpretation of data. LL and JL prepared the manuscript. All authors reviewed the manuscript.

### Conflict of interest statement

The authors declare that the research was conducted in the absence of any commercial or financial relationships that could be construed as a potential conflict of interest.
